# May-Thurner Syndrome: An Unusual Case of Unilateral Severe Deep Vein Thrombosis in a Middle-Aged Women

**DOI:** 10.7759/cureus.75621

**Published:** 2024-12-12

**Authors:** Ranjan Basu, Manohar Reddy, Ahmed Kaabneh, Aya Mohamedelamin Khidir Ahmed, Shrirang Bamne

**Affiliations:** 1 Internal Medicine, NMC Specialty Hospital, Abu Dhabi, ARE; 2 Radiology, NMC Specialty Hospital, Abu Dhabi, ARE; 3 Critical Care Medicine, NMC Specialty Hospital, Abu Dhabi, ARE

**Keywords:** deep vein thrombosis (dvt), iliac vein, iliac vein stent, inferior vena cava filter (ivcf), may-thurner syndrome

## Abstract

A 50-year-old female presented with a 10-day history of progressive swelling and pain in the left lower extremity, ultimately diagnosed with deep vein thrombosis (DVT) and May-Thurner Syndrome (MTS). Initial ultrasound indicated thrombosis involving the left external iliac, femoral, and popliteal veins, among others. Blood tests revealed normocytic anemia, but thrombophilia screening and other blood markers were normal. Computed tomography angiography (CTA) identified compression of the left common iliac vein (LCIV) by the right common iliac artery (RCIA), leading to thrombus formation. Initial management included anticoagulation with enoxaparin and iron supplementation. Interventional radiology was consulted, and an inferior vena cava (IVC) filter was placed. Through popliteal vein access, catheter-directed thrombolysis with Actilyse significantly reduced the thrombus burden. Venoplasty with a 12 mm balloon was performed to relieve the stenosis in the LCIV; however, recurrent stenosis required stent implantation. Post-stenting venography showed complete resolution of the stenosis and restored blood flow. This case illustrates the critical role of multimodal management in treating complex DVT with MTS, including anticoagulation, thrombolysis, and stent placement.

## Introduction

Compression of the left common iliac vein is independently associated with left-sided deep vein thrombosis [1]. In 1957, May and Thurner explained this phenomenon as a vascular condition caused by the compression of the left common iliac vein by the overlying right common iliac artery against a vertebral body [2]. This compression, combined with the pulsations of the adjacent artery, leads to gradual endothelial changes [3] and increases the risk of deep venous thrombosis in the left leg [4-6]. MTS, although an uncommon cause of DVT, should be considered in young, healthy individuals with unexplained lower extremity swelling and pain, particularly when traditional risk factors for DVT are absent.

This case report discusses a 50-year-old female patient from Sri Lanka with no significant thrombotic history who developed DVT in the left lower extremity secondary to MTS. She presented with progressive swelling and pain, prompting imaging studies that confirmed extensive thrombus formation and venous compression. The report highlights the diagnostic process, therapeutic intervention, and challenges of managing DVT complicated by MTS, underscoring the importance of a multidisciplinary approach for optimal outcomes.

## Case presentation

**Figure 1:**
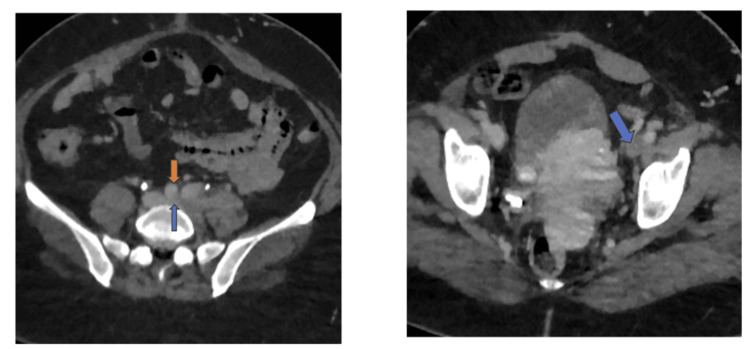
Left: Axial contrast CT showing compression of the left common iliac vein (blue arrow) and right common iliac artery (red arrow). The left common iliac vein appears flattened. Right: Axial CT showing thrombus in the left external iliac vein (blue arrow).

A 50-year-old woman from Sri Lanka was admitted to the hospital with a 10-day history of swelling and pain in her left lower limb. The swelling initially appeared in the thigh and progressively extended to the lower leg, accompanied by increased local skin tension and warmth. She reported no fever, shortness of breath, chest pain, cough, sputum production, hemoptysis, vision loss, or fainting. Her medical history was unremarkable, except for the use of bromocriptine tablets to manage hyperprolactinemia. She had no significant family or psychosocial history and no prior symptoms of chronic lower limb issues, even during her previous pregnancy. Deep vein ultrasound of the lower extremity in our hospital reported thrombosis of the left external iliac, common femoral vein, superficial femoral vein, deep femoral vein, popliteal vein, and port of left anterior tibial vein. The long saphenous vein was dilated and showed multiple septations, indicating evolving thrombosis. She had no prior history of surgery or catheterization. On physical examination, her heart rate was 82 bpm, and her blood pressure measured 124/76 mmHg. Evaluation of the left lower limb revealed swelling, edema, moderate tenderness, and pain while the right lower limb appeared normal. Peripheral pulsations were normal. Blood tests revealed normocytic anemia with Hb 8.7 g/dl, normal blood routine checkups, and normal liver and kidney functions. Her thrombophilia workup was normal. The patient had extensive deep vein thrombosis in the left lower limb extending proximally. CT venography was done as per a discussion with the interventional radiologist, as he thought it could show a more proximal extension of the thrombus, and to rule out any secondary issues that could predispose to thrombosis. Computed tomography angiography (CTA) revealed compression of the left common iliac vein (LCIV) by the right common iliac artery (RCIA), a narrowed LCIV lumen, and thrombus formation extending from the femoral vein to the left external iliac vein (Figure 1).

She was diagnosed with DVT along with MTS. She was treated with injection enoxaparin 100 mg subcutaneously bd, and injection ferric carboxymaltose 1000 mg IV and was referred to the interventional radiologist for further management.

Procedure by the interventional radiologist

**Figure 2:**
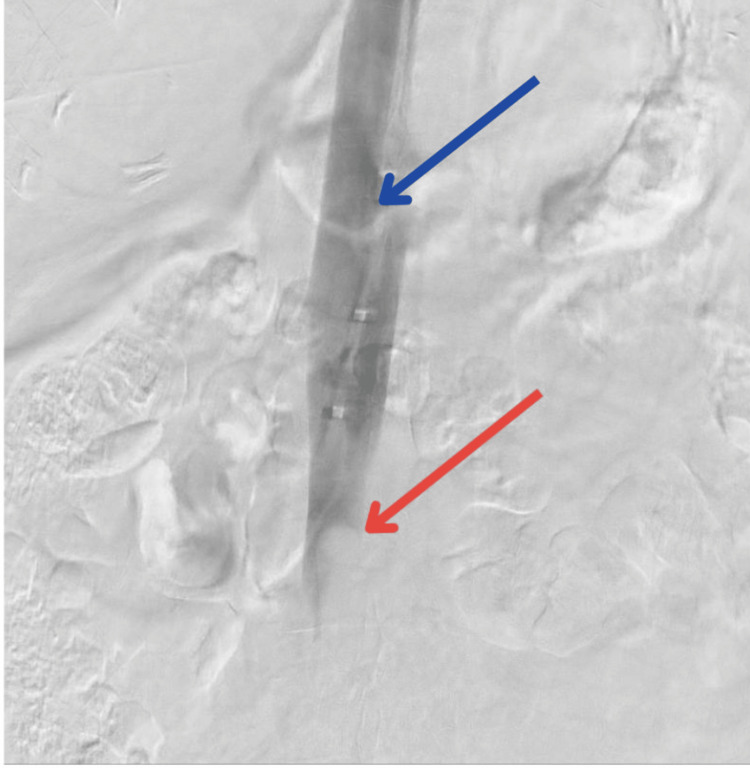
Inferior vena cava (blue arrow) and thrombus within the inferior vena cava (red arrow)

A CT review confirmed extensive DVT of the left lower limb and iliac vein, extending into the lower inferior vena cava (IVC) (Figure 2).

**Figure 3:**
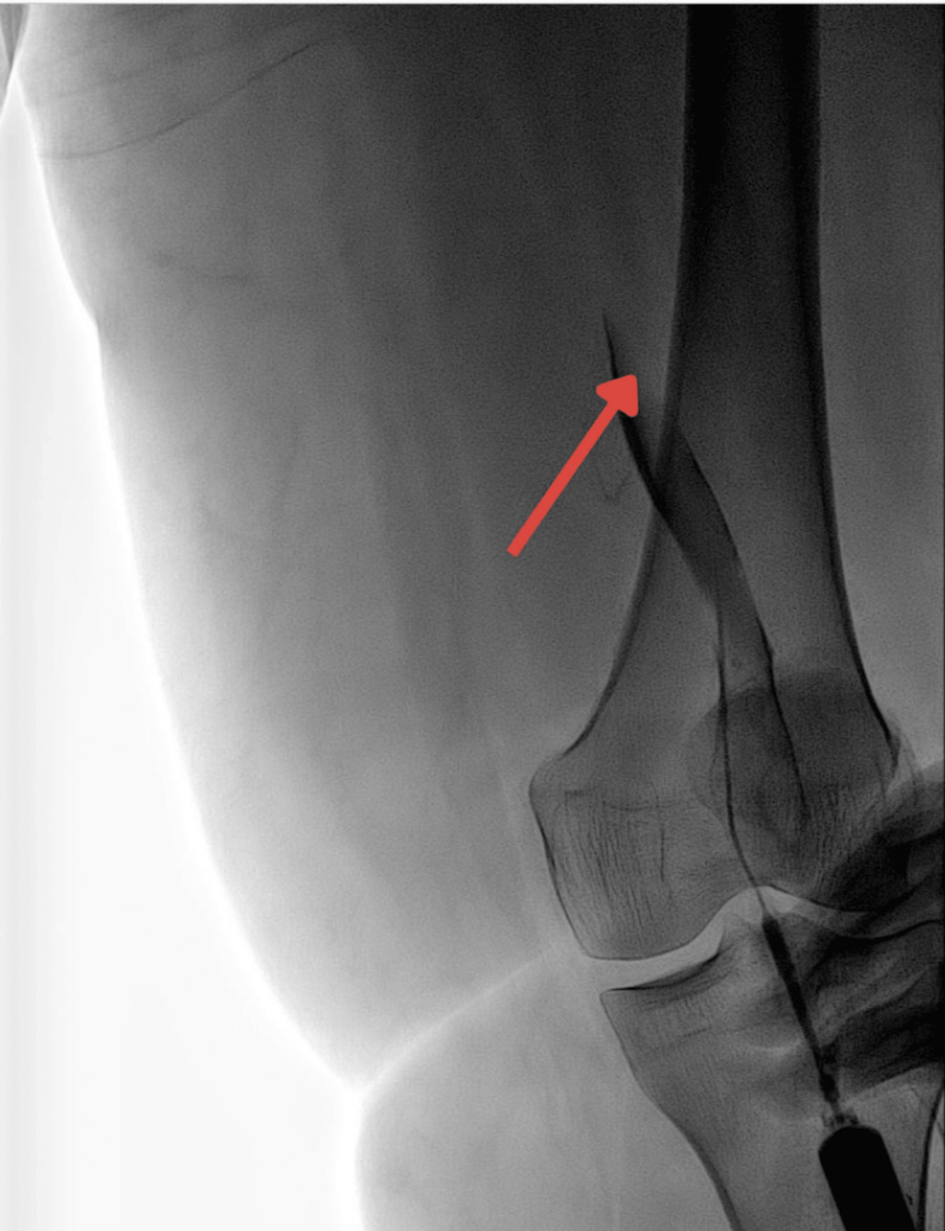
Popliteal access venogram showing occlusive DVT in the popliteal vein and superficial femoral vein DVT: deep vein thrombosis

A popliteal access venogram showing occlusive DVT in the popliteal vein and superficial femoral vein (Figure 3).

**Figure 4:**
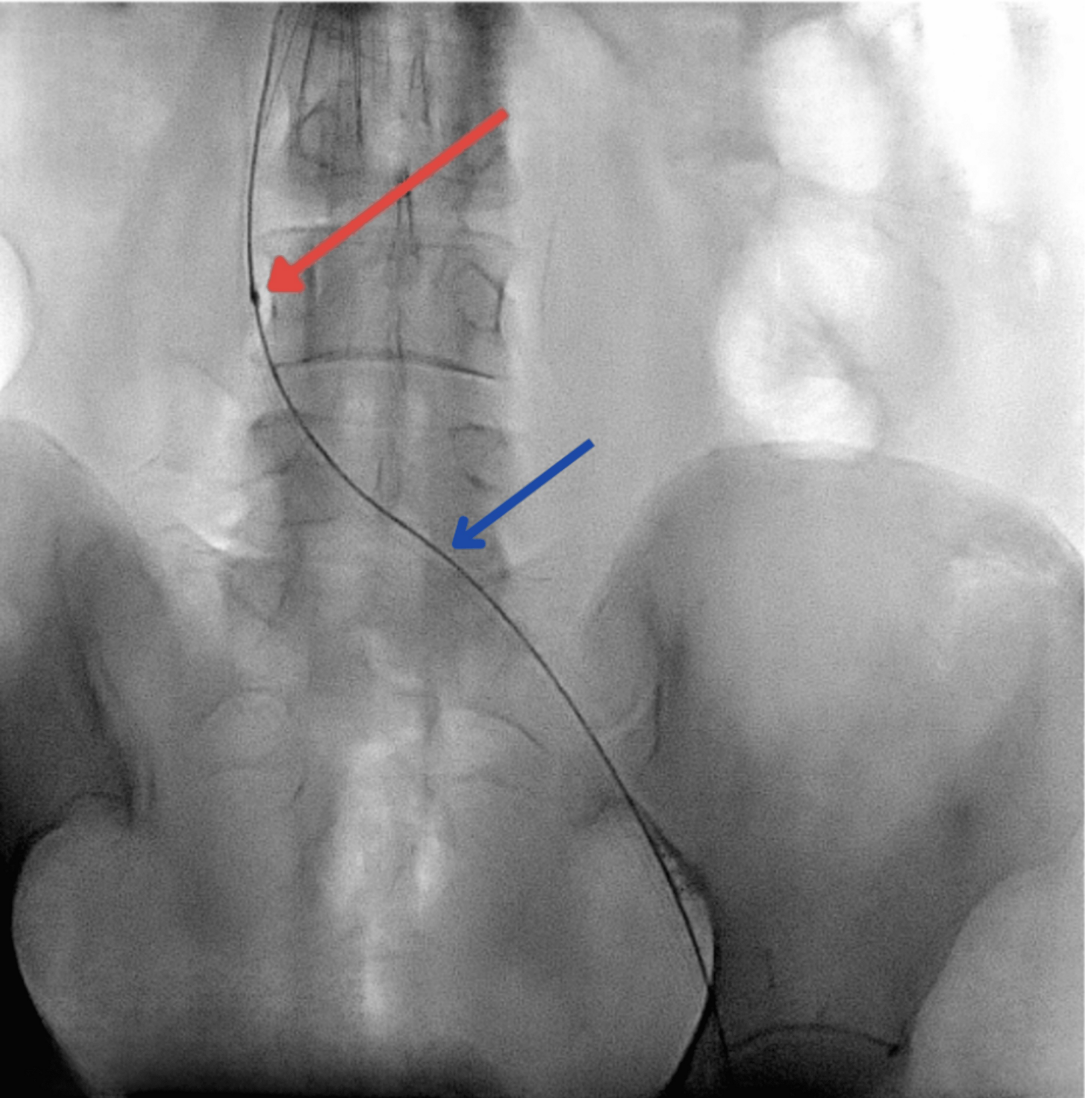
Infusion catheter traversing the DVT in the iliac veins with a tip place in the inferior vena cava (blue arrow) and a Cragg-McNamara valved infusion catheter (red arrow) DVT: deep vein thrombosis

An IVC filter was placed in the inferior vena cava via a jugular approach. The patient was then positioned prone, and a percutaneous puncture of the left popliteal vein was performed. The DVT, filling the femoral and iliac veins, was crossed using a 0.035 hydrophilic guidewire until access into the IVC was achieved. A fountain infusion catheter was advanced over the guidewire (Figure 4), with infusion markers covering the left iliac vein, common femoral vein (CFV), and proximal femoral vein.

**Figure 5:**
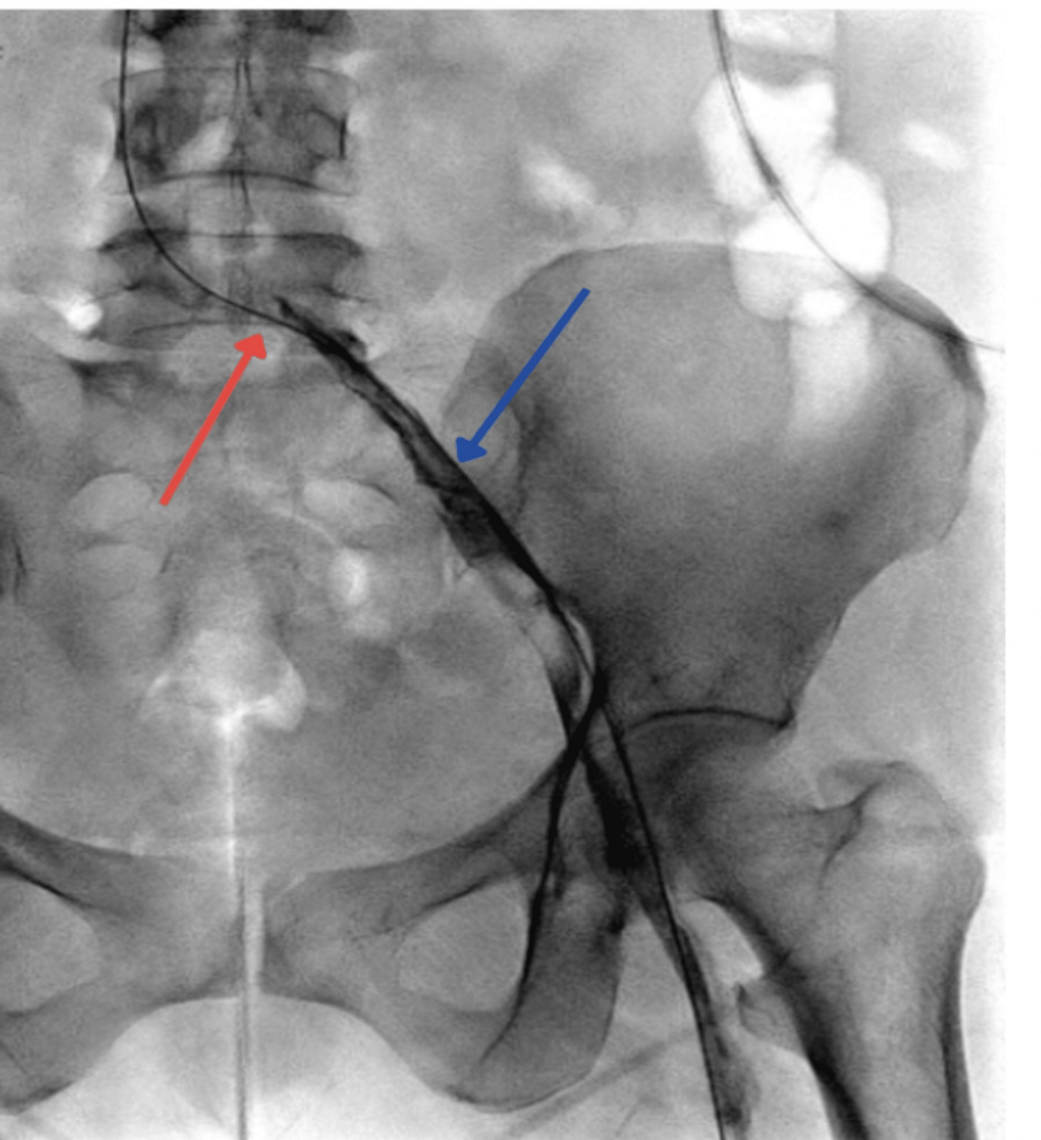
Post-lysis venogram confirming narrowing at the distal left common iliac vein (red arrow) and left leg vein (blue arrow)

A bolus of 8 mg Actilyse was administered through the infusion catheter, followed by a continuous infusion of 12 mg/day over 48 hours. Venography performed after 48 hours revealed a significant reduction in thrombus volume. However, a stenosis in the left common iliac vein was exposed (Figure 5).

**Figure 6:**
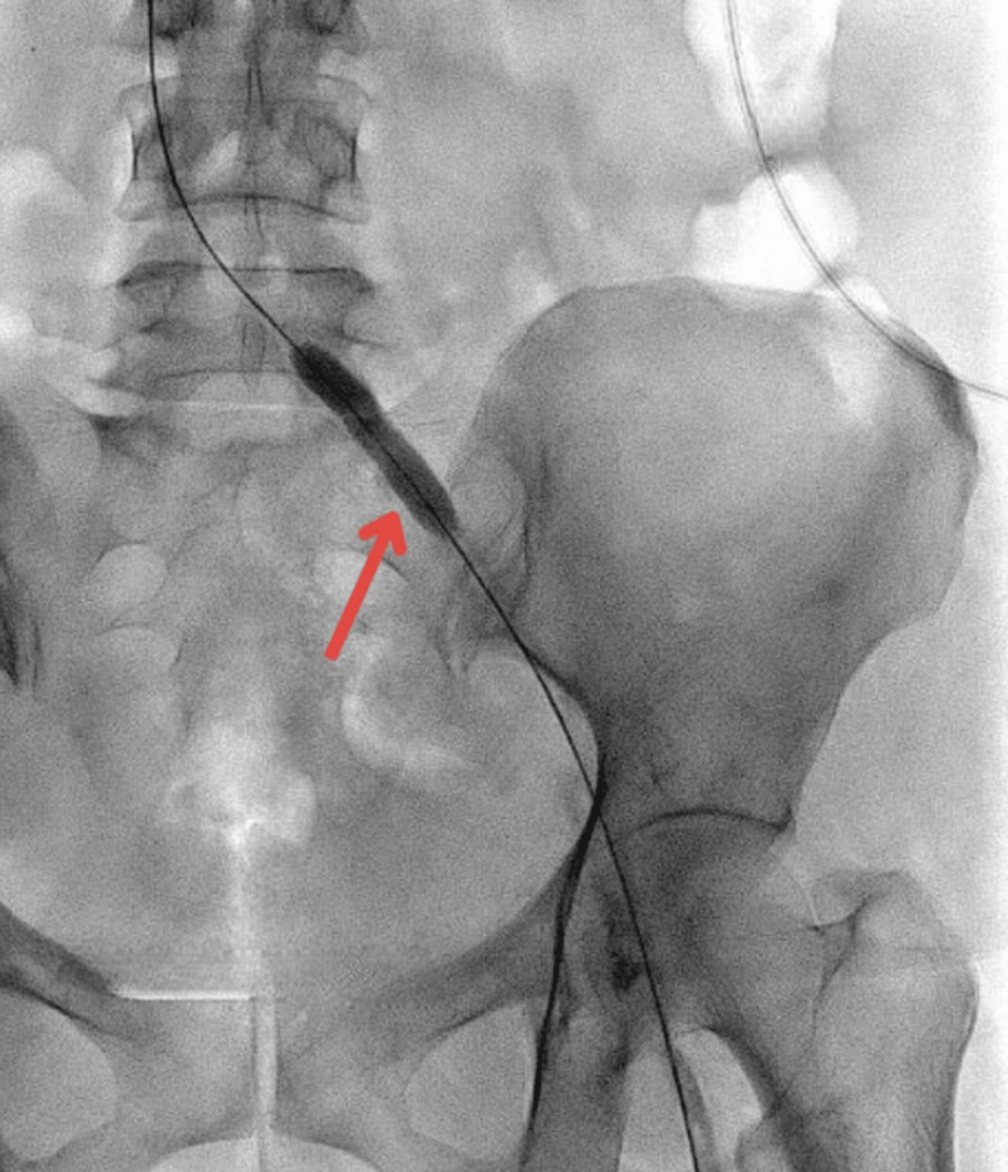
Balloon angioplasty tried with a 10x40 mm balloon (red arrow)

**Figure 7:**
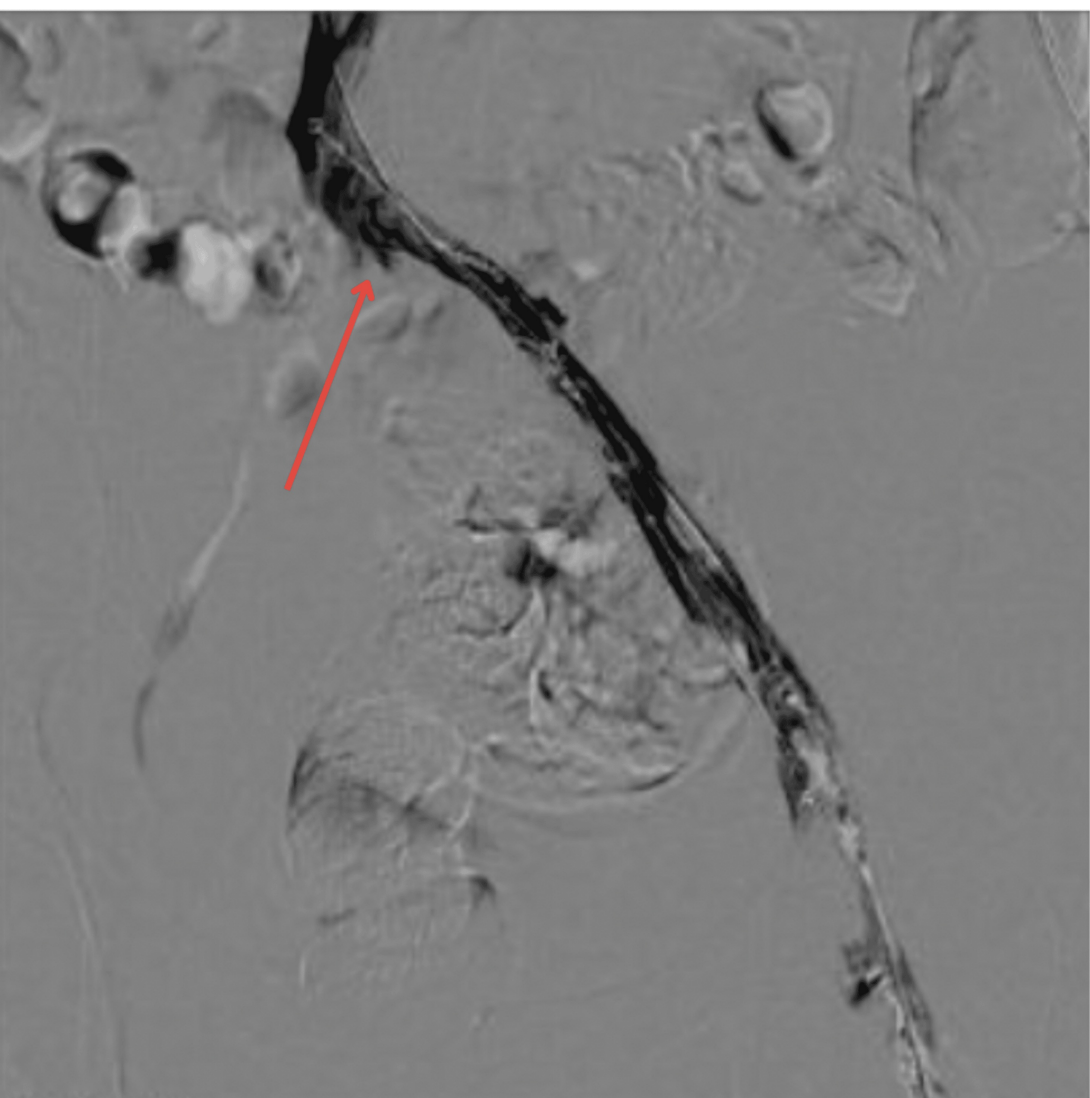
Flow restored post angioplasty but IVUS assessment confirmed persistent stenosis at the distal left common iliac vein (red arrow) IVUS: intravascular ultrasound

A 12 mm × 40 mm balloon was used to dilate the stenosis twice (Figure 6), but the stenosis recurred due to elastic recoil (Figure 7), as confirmed by intravascular ultrasound (IVUS).

**Figure 8:**
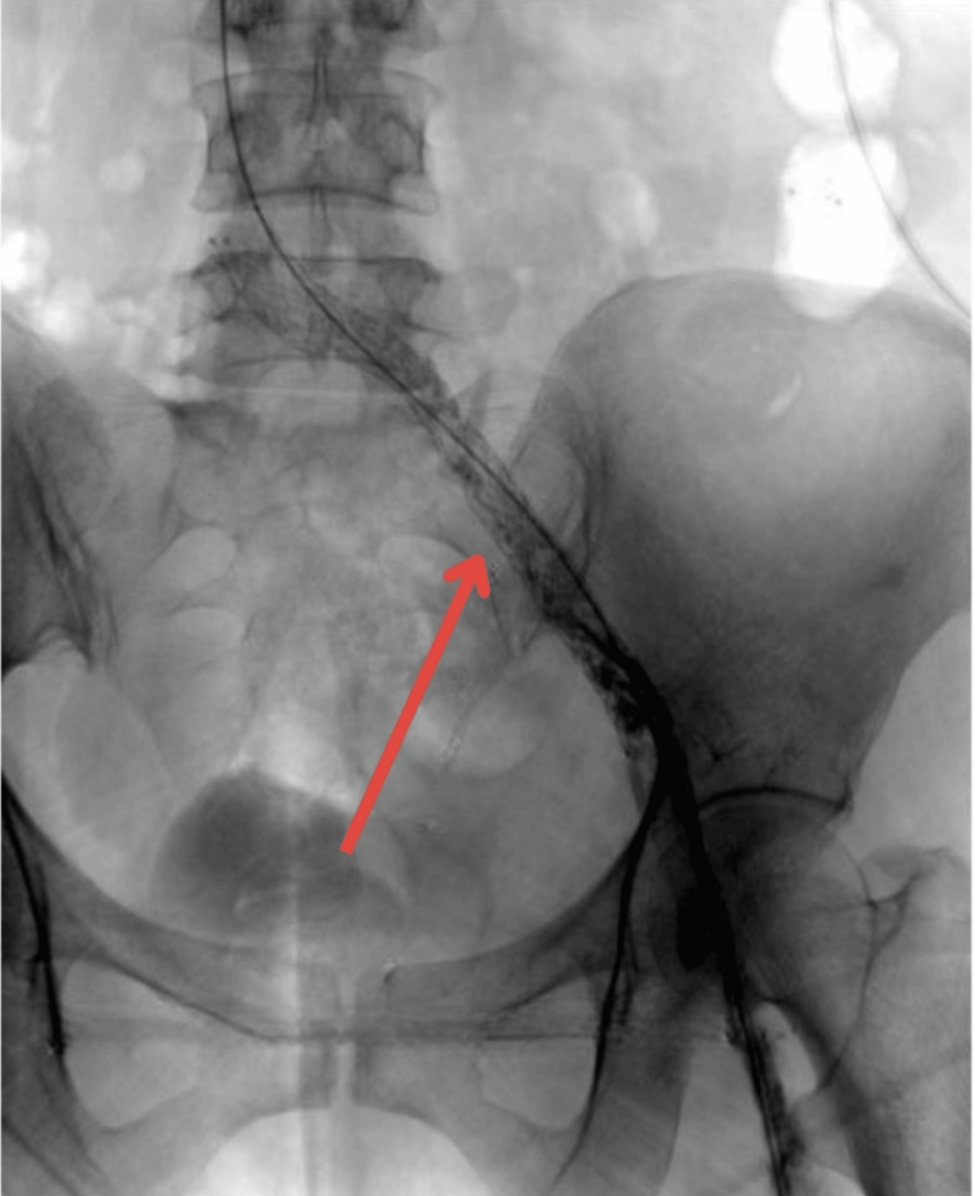
Venous stent deployed at the stenosis with a resolution of the narrowing (red arrow)

To address this, stent implantation was performed using a 12 mm × 145 mm stent in the left iliac vein. Post-stent venography showed complete resolution of the stenosis with unobstructed blood flow (Figure 8).

The patient was followed up one week after discharge as per protocol. Six weeks post-discharge, the inferior vena cava filter was removed. Subsequently, she was followed up every month, as she was on oral anticoagulation. A follow-up scan was planned six months post-discharge.

## Discussion

This case illustrates the complex nature of managing deep vein thrombosis (DVT) complicated by May-Thurner Syndrome (MTS), a condition where the right common iliac artery (RCIA) compresses the left common iliac vein (LCIV), leading to venous stasis and an increased risk of thrombus formation [7]. MTS, although an uncommon cause of DVT, should be considered in young, healthy individuals with unexplained lower extremity swelling and pain, particularly when traditional risk factors for DVT are absent.

In our patient, the absence of significant past medical history, including prior DVT or surgery, and the lack of risk factors, such as oral contraceptive use, suggest that MTS was the primary etiology for her thrombosis. Her clinical presentation, with swelling, pain, and increased skin tension in the left lower extremity, was consistent with acute DVT, but the underlying venous compression from MTS was revealed through imaging studies. Doppler ultrasound and CT angiography confirmed the diagnosis of DVT in the left iliac and femoral veins, and venous compression at the level of the LCIV was noted, characteristic of MTS.

The approach to treatment in this patient involved both medical and interventional strategies. Initial management with subcutaneous enoxaparin and intravenous iron supplementation addressed her thrombotic burden and anemia. Given the extensive thrombosis and the risk of further complications, including potential pulmonary embolism, she was referred for interventional radiology. The placement of an inferior vena cava (IVC) filter was crucial in preventing embolization of the clot to the lungs while catheter-directed thrombolysis using Actilyse was successful in reducing the thrombus burden.

However, despite thrombolysis, residual stenosis in the left common iliac vein persisted, and balloon venoplasty alone was insufficient due to elastic retraction. As a result, stent implantation was performed to maintain the patency of the vein and alleviate the compression from the RCIA. This intervention was successful, and subsequent venography confirmed complete relief of the stenosis, restoring unobstructed blood flow in the left iliac vein.

The management of DVT complicated by MTS underscores the importance of early and accurate diagnosis, as well as a tailored, multidisciplinary treatment approach. Anticoagulation therapy alone may not suffice in cases of extensive thrombosis or venous compression, and endovenous interventions, including thrombolysis and stenting, may be necessary to achieve optimal outcomes [8]. This case also highlights the role of advanced imaging techniques, such as CT angiography, in identifying underlying anatomical causes of DVT, such as MTS, which might otherwise be overlooked [9].

Similar cases have been reported in the literature [10], such as the case described by Yan Meng et al., in 2020, which has similar outcomes of the treatment. Further studies are needed to better understand the long-term outcomes of stenting in MTS-related DVT, as well as to evaluate the effectiveness of early intervention in preventing chronic venous insufficiency and post-thrombotic syndrome [11]. Early diagnosis and comprehensive management strategies, including both medical and interventional approaches, are critical in improving patient outcomes in such complex cases [12,13].

## Conclusions

This case underscores the importance of additional investigations in patients with extensive deep vein thrombosis (DVT) with proximal extension, as such investigations can help identify an underlying cause. It also highlights the value of a multidisciplinary approach in managing complex cases. Despite initial treatment with anticoagulation using enoxaparin and correction of our patient's anemia, the severity of the thrombosis necessitated further interventional management. A combination of IVC filter placement, catheter-directed thrombolysis, balloon angioplasty, and stenting successfully restored proper blood flow and resolved the stenosis. Post-procedure venography confirmed unobstructed blood flow and resolution of the stenosis. The patient responded well to the interventions and is expected to recover with appropriate follow-up care.

Further research is needed to better understand the long-term outcomes of stenting in May-Thurner Syndrome (MTS)-related DVT and to assess the effectiveness of early intervention in preventing chronic venous insufficiency and post-thrombotic syndrome. Early diagnosis and comprehensive management strategies, encompassing both medical and interventional approaches, are crucial for improving outcomes in such complex cases.
